# A Simple, Safe and Easy Mode of Preparing Oxygen

**Published:** 1866-01

**Authors:** 


A Simple, Safe and Easy Mode of Preparing Oxygen—is described by Dr. Reynolds. (*Brit. Phot. Jour.*) It is based on the decomposition of hypochlorite of lime in the presence of peroxide of cobalt. The former he obtains from bleaching powder, or "chloride of lime," by extracting with cold water. The peroxide of cobalt is prepared by precipitating a solution of nitrate of cobalt with this solution, until a precipitate ceases to fall, and washing with water. The peroxide produced from one ounce of the nitrate is added to a concentrated solution of two pounds of bleaching powder (in one quart of water) in a generating bottle, and the evolution of oxygen proceeds at once "with great regularity, but the gas comes off in a very rapid stream if the generating vessel be immersed in warm water at a temperature of about 100° or 120° Fahr." The oxygen thus obtained is said to be very pure. The process moreover is economical, for *all* the oxygen of the hypochlorite of lime is given off, thus:

